# Clinical and prognostic significance of concomitant autoimmune disease in patients with myelofibrosis

**DOI:** 10.1007/s00277-026-07128-4

**Published:** 2026-06-26

**Authors:** Alessandro Costa, Olga Mulas, Marianna Greco, Martina Piras, Gabriele Accossu, Federica Pilo, Giovanni Caocci

**Affiliations:** 1 Hematology, Department of Medical Sciences and Public Health, University of Cagliari, Cagliari, 09121 Italy; 2 Hematology and BMT Unit, “A. Businco” Hospital, ARNAS Brotzu, Cagliari, 09121 Italy; 3 General Medicine Unit, Hospital San Michele, ARNAS “G. Brotzu, Cagliari, 09121 Italy

**Keywords:** Autoimmune disease, Myeloproliferative neoplasms, Myelofibrosis, Outcome, Transformation

## Abstract

There is growing interest in the association between autoimmune diseases (AID) and myeloproliferative neoplasms (MPN), yet the specific clinical and prognostic implications of AID in myelofibrosis (MF) remain insufficiently characterized. A retrospective analysis was conducted in patients diagnosed with primary MF (PMF) or secondary MF (SMF). Concomitant AID diagnoses were identified through medical record review and included only cases with documented clinical diagnosis. A total of 135 patients with MF were analyzed, including 90 (66.7%) with PMF and 45 (33.3%) with SMF. At diagnosis, 31 AID diagnoses were recorded in 29 patients (21.5%), of which 54.8% were organ-specific and 45.2% systemic. AID was more frequent in female patients (*p* = 0.005). At a median follow-up of 37.3 months, 3-year overall survival (OS) was 78%, with inferior OS in patients with systemic AID (61%; *p* = 0.028) compared to those without AID (79%) or with organ-specific AID (88%). In multivariable analysis, systemic AID (HR 2.16, p = 0.043), anemia (HR 1.79, *p* = 0.041), age (HR 1.08, *p* < 0.001), and JAK-inhibitor therapy (HR 0.57, *p* = 0.023) independently predicted mortality. Severe infections and thrombotic events were more frequent in AID patients (26% vs 9.3%, *p* = 0.0082), with significance retained for infections alone (*p* = 0.016). The presence of AID did not affect the rate of leukemic transformation. Systemic AID identifies a subset of MF patients with worse survival and increased susceptibility to infections, supporting intensified surveillance and tailored management strategies in this population.

**Supplementary Information:**

The online version contains supplementary material available at https://doi.org/10.1007/s00277-026-07128-4.

## Introduction

Among *BCR::ABL1*-negative myeloproliferative neoplasms (MPNs), myelofibrosis (MF) represents the most aggressive disease, marked by altered bone marrow function and fibrosis [1]. The clinical burden is substantial and frequently includes fatigue, constitutional symptoms, and splenomegaly-related manifestations such as early satiety and abdominal discomfort [2]. Since 2016, primary myelofibrosis (PMF) has been subclassified into prefibrotic and overt fibrotic disease, each with distinct clinical and prognostic implications. Approximately 15% of patients with essential thrombocythemia (ET) or polycythemia vera (PV) progress to secondary MF (post-ET/post-PV MF), which shares clinical and therapeutic features with PMF. In recent years, molecular insights into MF have expanded beyond the canonical driver mutations to include additional somatic mutations in myeloid-associated genes such as *ASXL1*, *DNMT3A*, *EZH2*, *SRSF2*, and *U2AF1*, found in 40–60% of cases [3]. Consequently, recognition of clinical and genetic prognostic factors has improved markedly over the past decades, enabling refinement of prognostic scoring systems and therapeutic decision-making pathways.

There is growing interest in the role of autoimmunity in myeloid neoplasms [4]. Although the association between autoimmune diseases (AID) and lymphoid malignancies is well recognized [5, 6], autoimmune phenomena have also been documented in myeloid malignancies. For instance, a concomitant AID has been reported in 16% of 15.277 patients with myelodysplastic syndromes (MDS) [7] and in 36% of 131 patients with chronic myelomonocytic leukemia (CMML) [8]. In MPNs, autoimmune conditions such as autoimmune thyroiditis, rheumatoid arthritis (RA), systemic lupus erythematosus (SLE), vasculitis, autoimmune hemolytic anemia (AIHA) and immune thrombocytopenia (ITP) have also been reported, although their prevalence and clinical significance remain less well defined [7, 9–11]. A large population-based study encompassing over 11.000 MPN patients and 43.000 matched controls reported a modest but statistically significant increased risk of MPN among individuals with pre-existing AID [11]. In a single-center cohort, 8% of MPN patients presented with concomitant AID; this subgroup exhibited younger age at diagnosis, female predominance, higher frequency of splenomegaly, and an elevated risk of disease progression [12]. More recently, data from 1.968 patients with ET or PV, 6% of whom had a prior AID diagnosis, showed no significant impact of AID on thrombosis or transformation risk but identified an increased incidence of secondary malignancies, independent of age, sex, and prior cancer history [13]. Notable advances were made by Elessa et al. [14], who analyzed the mutational landscape of 1.541 MPN patients, including 95 (6%) with AID, reporting a higher prevalence of *TET2* mutations in MF patients with AID, while no differences were observed in driver mutation frequencies or overall mutational burden between groups.

Despite growing interest, most available data on AID in MPN are derived from mixed cohorts or focus primarily on ET and PV, with limited evaluation of individual disease subtypes. In particular, the clinical, prognostic, and therapeutic implications of coexisting AID in MF remain poorly defined. To address this gap, we aimed to investigate the impact of AID in patients with MF, with the hypothesis that this comorbidity might independently influence disease presentation, progression, and outcomes.

## Materials and methods

### Study cohort

We performed a retrospective single-center analysis of patients diagnosed with MF and managed at our oncological referral institution from March 2002 to July 2025. Inclusion required a diagnosis of PMF or post–PV/ET MF, according to the 2022 WHO criteria [15], age ≥18 years, and availability of complete morphological, cytogenetic, and MPN driver mutation profiling. Patients with missing essential diagnostic or follow-up information were excluded from the study cohort at baseline. For secondary variables, analyses were performed using a complete-case approach without data imputation.

## Data collected

Baseline clinical, hematologic, cytogenetic, and molecular variables, along with treatment history and outcome data, were extracted through a comprehensive review of institutional medical records. Prognostic assessment at diagnosis was performed using the Dynamic International Prognostic Scoring System (DIPSS) for patients with PMF [16] and the MYSEC-PM score for patients with secondary MF [17]. Longitudinal data included adverse clinical events, including infectious complications, cardiovascular events, and diagnosis of secondary neoplasms. Adverse events were graded according to the Common Terminology Criteria for Adverse Events (CTCAE), version 5.0. Survival status was updated at the last follow-up. Analyses and results are reported in accordance with the STROBE guidelines (**Supplementary Table ****1**) [18].

## Assessment of concomitant AID

Concomitant AID were identified through retrospective review of medical records and included only cases with a confirmed clinical diagnosis at baseline or during follow-up. In patients with PMF, the autoimmune disorder was required to precede the diagnosis of MF; in those with post-PV/ET MF, it had to precede the diagnosis of the antecedent MPN. As no universally accepted classification of AID is currently available, we adopted a pragmatic distinction between systemic (e.g., connective tissue diseases, systemic vasculitis) and organ-specific (e.g., autoimmune thyroiditis, autoimmune cytopenias), as previously proposed [19]. Isolated serologic abnormalities and non-autoimmune inflammatory conditions were excluded. To minimize potential bias, cases fulfilling criteria for autoimmune myelofibrosis were excluded after integrated morphologic, clinical, and serological assessment. In triple-negative MF cases, the distinction between clonal MF and autoimmune/reactive marrow fibrosis was based on integrated clinicopathologic review, including bone marrow morphology, clinical phenotype, cytogenetic findings, and NGS-derived myeloid mutation data when available.

### Statistical analysis

Continuous variables were reported as median and interquartile range (IQR), while categorical variables as frequency and percentage. Comparisons among groups were performed using the Chi-square test or Fisher’s exact test for categorical variables, and the Mann-Whitney U test for continuous variables, according to normality assumptions assessed by the Shapiro–Wilk test. Overall survival (OS) was defined as the time from diagnosis of PMF or post-ET/post-PV MF to death from any cause or last follow-up. Leukemia-free survival (LFS) was defined as the time from diagnosis to leukemic transformation, with death without transformation treated as a competing event. Cumulative incidence functions were estimated within a competing-risk framework, considering adverse clinical events, including infections, cardiovascular events, and secondary malignancies, as events of interest, and unrelated death as a competing event. Differences between cumulative incidence functions were assessed using Gray’s test. Survival analysis was performed using Kaplan–Meier curves, with differences assessed by the log-rank test. Hazard ratios (HRs) with 95% confidence intervals (CIs) for survival-associated factors were estimated using univariable and multivariable Cox proportional hazards regression models. A level of *p* < 0.05 was used to define statistical significance in all tests. Statistical analyses were performed using R software (R Core Team, 2021), version 2025.05.0 + 496 (R Foundation for Statistical Computing, Vienna, Austria).

## Results

A total of 140 patients with MF were collected, of whom 135 were included in the analysis and 5 were excluded for not meeting the inclusion criteria (**Supplementary Fig. ****S1**). The cohort comprised 90 (66.7%) patients with PMF and 45 (33.3%) with secondary MF. The median age at diagnosis was 68.0 years (IQR: 61.0–75.0), and 82 patients (60.7%) were male. Anemia was present in 55 patients (40.7%) at diagnosis, with 25 (18.5%) exhibiting hemoglobin levels < 8 g/dL. The median leukocyte count was 8.7 × 10⁹/L (IQR, 5.2–13.4), the median platelet count was 318 × 10⁹/L (IQR: 150–539), and the median lactate dehydrogenase (LDH) was 477 U/L (IQR: 299–817). Palpable splenomegaly was observed in 97 patients (71.9%). Other baseline demographic and clinical characteristics are detailed in Table 1.

**Table 1:** Demographic and clinical characteristics

Variables	Total, *n* = 135	No AID, *n* = 106	AID, *n* = 29	*p*
Female sex, *n* (%)	53 (39.3)	35 (33.0)	18 (62.1)	0.005*
Age, median years (IQR)	68.0 (61.0–75.0)	68 (61–74)	69 (64–77)	0.287
PMF, *n* (%)	90 (67.2)	73 (68.9)	17 (58.6)	0.374
Overt PMF	72 (53.3)	57 (53.8)	15 (51.7)	0.506
Prefibrotic PMF	18 (13.3)	16 (15.1)	2 (6.9)	
SMF, *n* (%)	45 (33.3)	33 (31.1)	12 (41.4)	0.273
Post-ET MF	32 (23.7)	21 (19.8)	11 (37.9)	0.051
Post-PV MF	13 (9.7)	12 (11.3)	1 (3.4)	
Hb, g/dL, median (IQR)	10.4 (8.8–12.1)	10.5 (8.9–12.2)	10.1 (8.6–12.1)	0.825
Hb > 10 g/dL	80 (59.3)	64 (60.4)	16 (55.2)	0.878
Hb 8–10 g/dL	30 (22.2)	23 (21.7)	7 (24.1)	
Hb < 8 g/dL	25 (18.5)	19 (17.9)	6 (20.7)	
WBCs, x10^9^/L, median (IQR)	8.7 (5.2–13.4)	8.74 (5.3–13.4)	9.1 (4.6–19.5)	0.871
Hematocrit, % (IQR)	33.1 (28.4–37.8)	33.1 (28.5–37.5)	33.2 (28.4–38.5)	0.921
PLTs, x10^9^/L, median (IQR)	318 (150–539)	314 (153–552)	331 (148–507)	0.888
LDH, median U/I (IQR)	477 (299–817)	490 (312–773)	442 (254–836)	0.813
Palpable splenomegaly, *n* (%)	97 (71.9)	79 (74.5)	18 (62.1)	0.276
Abnormal cytogenetic, *n* (%)	29 (21.5)	21 (19.8)	8 (27.6)	0.444
Driver mutations, *n* (%)				0.905
*JAK2* V617F mut	82 (60.7)	65 (61.3)	17 (58.6)	
*CALR* mut	27 (20.0)	20 (18.9)	7 (24.1)	
*MPL* mut	7 (5.2)	6 (5.7)	1 (3.4)	
Triple-negative	19 (14.1)	15 (14.2)	4 (13.8)†	
DIPSS, *n* (%)				0.599
Low-risk	6 (4.4)	6 (5.7)	0	
Intermediate-1	27 (20.0)	21 (19.8)	6 (20.7)	
Intermediate-2	46 (34.1)	38 (35.8)	8 (27.6)	
High risk	11 (8.1)	8 (7.5)	3 (10.3)	
MYSEC-PM, *n* (%)				0.427
Low-risk	6 (4.4)	5 (4.7)	1 (3.4)	
Intermediate-1	22 (16.3)	17 (16.0)	5 (17.2)	
Intermediate-2	11 (8.1)	6 (5.7)	5 (17.2)	
High risk	6 (4.4)	5 (4.7)	1 (3.4)	
CCI, *n* (%)				0.295
0–1	22 (16.3)	18 (17.0)	4 (13.8)	
2–4	89 (65.9)	72 (67.9)	17 (58.6)	
>4	24 (17.8)	16 (15.1)	8 (27.6)	

*Statistically significant differences

†All 4 triple-negative patients with concomitant AID harbored additional myeloid-associated mutations by NGS: *DNMT3A*, *TET2*/*SF3B1*, *ASXL1*/*CBL*, and *U2AF1*, respectively. AID, autoimmune disease; DIPSS, Dynamic International Prognostic Scoring System; ET, essential thrombocythemia; Hb, hemoglobin; IQR, interquartile range; MYSEC, Myelofibrosis Secondary to PV and ET-Prognostic Model; PLT, platelets; PMF, primary MF; PV, polycythemia vera; SMF, secondary MF; WBC, white blood cells

Driver mutation analysis showed *JAK2* V617F in 82 patients (60.7%), *CALR* mutations in 27 (20.0%), and *MPL* mutations in 7 (5.2%). Nineteen patients (14.1%) were triple-negative. Among 119 evaluable patients, cytogenetic abnormalities were detected in 29 (24.4%), most frequently del(13q) (*n* = 5), del(20q) (*n* = 4), + 8 (*n* = 5), + 9 (*n* = 5), unbalanced translocations (*n* = 3), del(11q) (*n* = 2), and complex karyotypes (*n* = 2). Less frequent findings included del(9q), del(7q), del(16q), + 6, and − 7 (*n* = 1 each). Next-generation sequencing (NGS) data were available for 24 patients. The most frequently mutated genes were *TET2* (*n* = 5), *ASXL1* (*n* = 4), *SRSF2* (*n* = 3), *BCOR* (*n* = 3), *IDH1* and *NF1* (*n* = 2 each). Single occurrences were observed for *DNMT3A*, *SF3B1*, *KIT*, *SH2B3*, *U2AF1*, *TP53*, *NRAS*, *RUNX1*, *CBL*, *STAG2*, *KRAS*, *ZRSR2* and *CSF3R*.

Overall, 112 patients (83.0%) reported at least one comorbidity at diagnosis. Cardiovascular conditions were the most common (67.4%), with arterial hypertension being the leading comorbidity (51.9%), followed by atrial fibrillation (8.1%) and ischemic stroke or transient ischemic attack (TIA) (5.2%). Type 2 diabetes mellitus was present in 18 patients (13.3%), and 29 (21.5%) had dyslipidemia. A history of thrombotic events was reported in 5 patients (3.7%).

### Spectrum of concomitant AID and comparison between cohorts

A total of 31 AID were identified in 29 patients (21.5%) at the time of diagnosis. The organ involvement is summarized in Table 2. Seventeen events (54.8%) were classified as organ-specific, including autoimmune thyroiditis (*n* = 11), type 1 diabetes mellitus (*n* = 1), warm AIHA (*n* = 2), ulcerative colitis (*n* = 2) and atrophic gastritis (*n* = 1). Fourteen events (45.2%) were classified as systemic AID, including RA (*n* = 5), antiphospholipid syndrome (*n* = 2), SLE (*n* = 2), psoriasis/psoriatic arthritis (*n* = 3), mixed connective tissue disease (*n* = 1), and autoimmune vasculitis (*n* = 1). Two patients had two concomitant organ-specific AID. Endocrine and rheumatologic AID were most common. In all patients included in the baseline AID cohort, diagnosis of AID preceded the diagnosis of MF. The median interval between AID diagnosis and MF diagnosis was 67 months (IQR, 12–90). At diagnosis, 12 patients (41.4%) were on replacement therapy (levothyroxine, insulin, vitamin B12 replacement), 3 (10.3%) were receiving disease-modifying antirheumatic drugs (DMARDs) including methotrexate, hydroxychloroquine, and sulfasalazine (each *n* = 1), 7 (24.1%) were receiving corticosteroids, and one patient (3.4%) was receiving nonsteroidal anti-inflammatory drugs (NSAIDs).

**Table 2:** Spectrum of concomitant AID at diagnosis

Category	Diagnosis	*n* (%)
Endocrine AID	Autoimmune thyroiditis	11 (34.4)
	Type 1 diabetes mellitus	1 (3.1)
Rheumatologic AID	Rheumatoid arthritis	5 (15.6)
	APS	2 (6.3)
	SLE	2 (6.3)
	Psoriatic arthritis	1 (3.1)
	Mixed connective tissue disease	1 (3.1)
	Autoimmune vasculitis	1 (3.1)
Hematologic AID	Warm AIHA	2 (6.3)
Cutaneous AID	Psoriasis	2 (6.3)
Gastroenterological AID	Ulcerative colitis	2 (6.3)
	Atrophic gastritis	1 (3.1)

AID, autoimmune diseases; APS, antiphospholipid syndrome; SLE, systemic lupus erythematosus

A history of AID was significantly more frequent among female patients (*p* = 0.005). Median age at diagnosis did not differ significantly between patients with and without AID (*p* = 0.287). Routine blood counts were also comparable (Table 1). Cardiovascular comorbidities at diagnosis (*p* = 0.717) and prior secondary malignancies (*p* = 1.0) did not differ significantly. The distribution of primary versus secondary MF was similar between groups (*p* = 0.273), as was the ratio of prefibrotic to overt PMF (*p* = 0.506). A trend toward a higher prevalence of post-ET MF was noted among patients with AID (*p* = 0.051). Driver mutation frequencies did not differ (*p* = 0.905), nor did risk stratification by DIPSS (*p* = 0.599) or MYSEC-PM (*p* = 0.427). No statistically significant differences were observed between patients with systemic versus organ-specific AID in terms of clinical and laboratory characteristics (**Supplementary Table ****2**).

Overall, 117 patients (86.7%) received at least one line of therapy for MF (**Supplementary Table ****3**). In the frontline setting, 50 patients (37.0%) were treated with hydroxyurea and 50 (37.0%) with a JAK-inhibitor. Seventeen patients (12.6%) required second-line therapy, most commonly a JAK-inhibitor (9 patients, 6.7%). No significant differences were observed between cohorts in terms of number of therapy lines (*p* = 0.638) or treatment distribution across lines.

During follow-up, 5 patients (3.7%) without prior AID developed new autoimmune manifestations, including four females and one male. These events comprised Coombs-positive hemolytic anemia, autoimmune thrombocytopenia with anti-GpIa/IIa antibodies, autoimmune gastritis, primary biliary cholangitis, and autoimmune hepatitis. The median time from MF diagnosis to incident AID onset was 30 months (IQR, 14–52). At the time of AID development, 2 of these 5 patients were receiving JAK-inhibitor therapy.

## Survival analysis and comparison between patients with and without history of AID

**Fig. 1:**
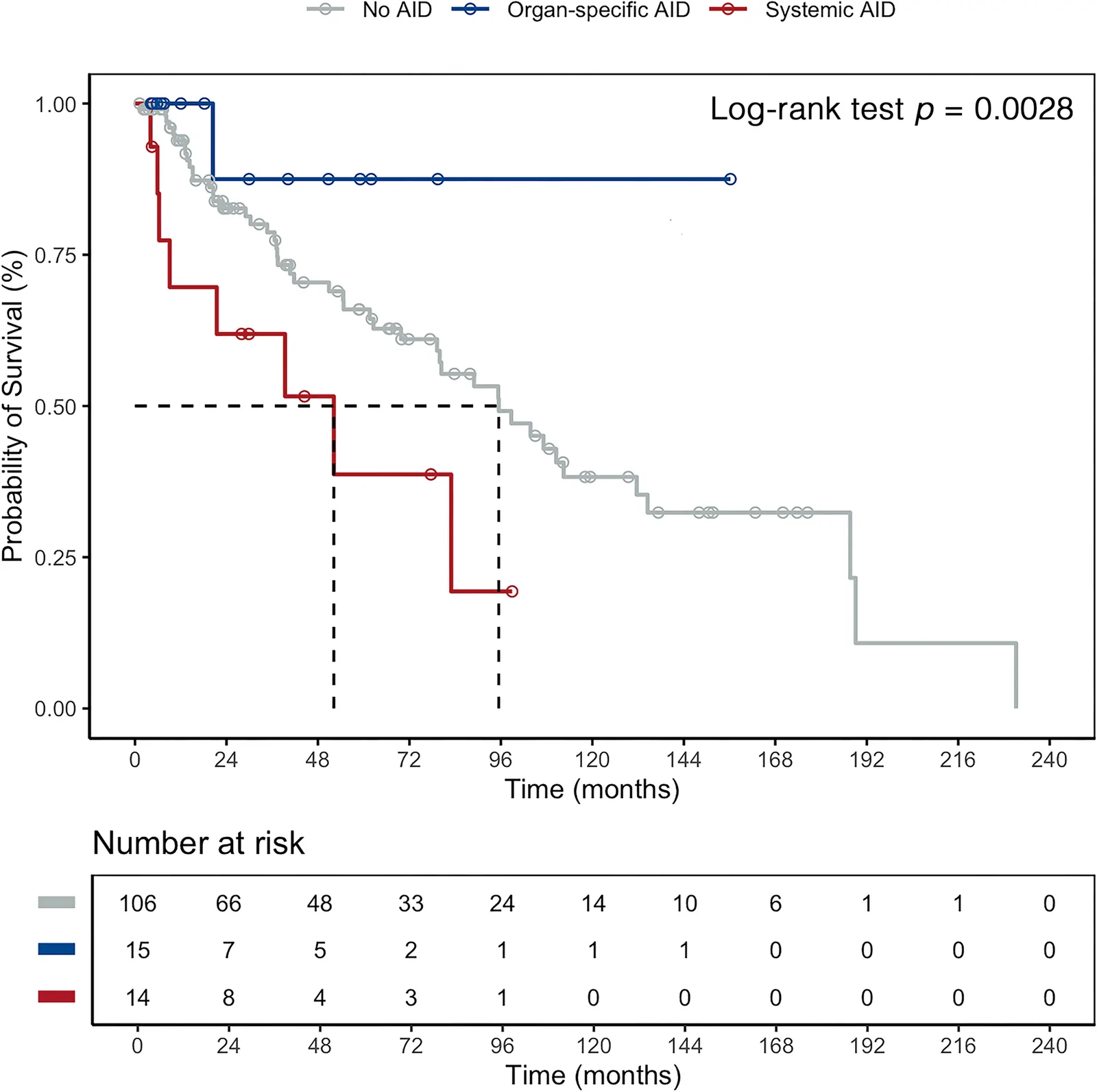
Outcomes of MF patients without pre-existing AID compared to patients with a history of organ-specific and systemic AID. Median OS did not differ significantly between patients with (*n* = 29) and without (*n* = 106) any history of AID (83 vs. 95 months, *p* = 0.6). However, when stratified by AID subtype, patients with systemic AID (*n* = 14) had significantly inferior 3-year OS (61%) compared to those with organ-specific AID (*n* = 15, 88%) and those without AID (79%) (*p* = 0.028). Abbreviations: AID, autoimmune disease; OS, overall survival

At data cutoff, 56 deaths (41.5%) had occurred. After a median follow-up of 37.3 months (IQR: 13.5–79.7), 3-year OS was 78% (95% CI, 70–86). Median OS was 95 months (95% CI, 79–134) in patients without a history of AID, and 83 months (95% CI, 52–not estimable [NE]) in those with AID, with no statistically significant difference between groups (*p* = 0.6). However, when stratified by AID subtype, 3-year OS was 79% (95% CI, 71–88) in patients without AID, 88% (95% CI, 67–100) in those with organ-specific AID, and 61% (95% CI, 40–95) in those with systemic AID (*p* = 0.028) (Fig. 1).

Given the impact of systemic forms on survival, a Cox regression model was constructed including systemic AID, anemia, age, female sex, splenomegaly and therapy with JAK-inhibitors (Table 3). In univariate analysis, systemic AID (HR 2.4; 95% CI, 1.1–5.1; *p* = 0.026), anemia (HR 1.9; 95% CI, 1.1–3.3; *p* = 0.018), age (HR per year 1.1; 95% CI, 1.0–1.1; *p* < 0.001) were significantly associated with reduced survival, while therapy with JAK-inhibitors was associated with better survival (HR 0.57, 95% CI 0.3–0.9, *p* = 0.023); these variables remained independent predictors in multivariate analysis.

**Table 3:** Cox regression model for overall survival (OS)

Variables	Univariate analysis			Multivariate analysis		
	HR	95% CI	*p*	HR	95% CI	*p*
History of systemic AID	2.4	1.1–5.1	0.026*	2.16	1.03–4.76	0.043*
Baseline anemia	1.9	1.1–3.3	0.018*	1.79	1.02–3.12	0.041*
Age (per additional year)	1.1	1-1.1	< 0.001*	1.08	1.04–1.12	< 0.001*
Female sex	0.58	0.3-1	0.069	0.57	0.31–1.05	0.07
Presence of splenomegaly	0.64	0.3–1.1	0.12	0.73	0.4–1.34	0.310
Treatment with a JAK-inhibitor	0.57	0.3–0.9	0.023*	0.53	0.3–0.8	0.038*

*Statistically significant differences

AID, autoimmune disease; CI, confidence interval; HR, hazard ratio

Leukemic transformation occurred in 11 patients (8.1%), with a 3-year cumulative incidence of 8.7% (95% CI, 4.1–15). No significant differences were observed between patients with and without AID, respectively 3.4% (95% CI, 0.24–15) vs. 9.8% (95% CI, 4.4–18, *p* = 0.50). When stratified by AID subtype, the rate was 9.8% (95% CI, 4.4–18) in patients without AID and 7.1% (95% CI, 0.39–28) in those with systemic AID, while no progression was recorded in those with organ-specific AID (*p* = 0.6).

## Incidence and comparison of adverse events in MF patients with and without AID

**Fig. 2:**
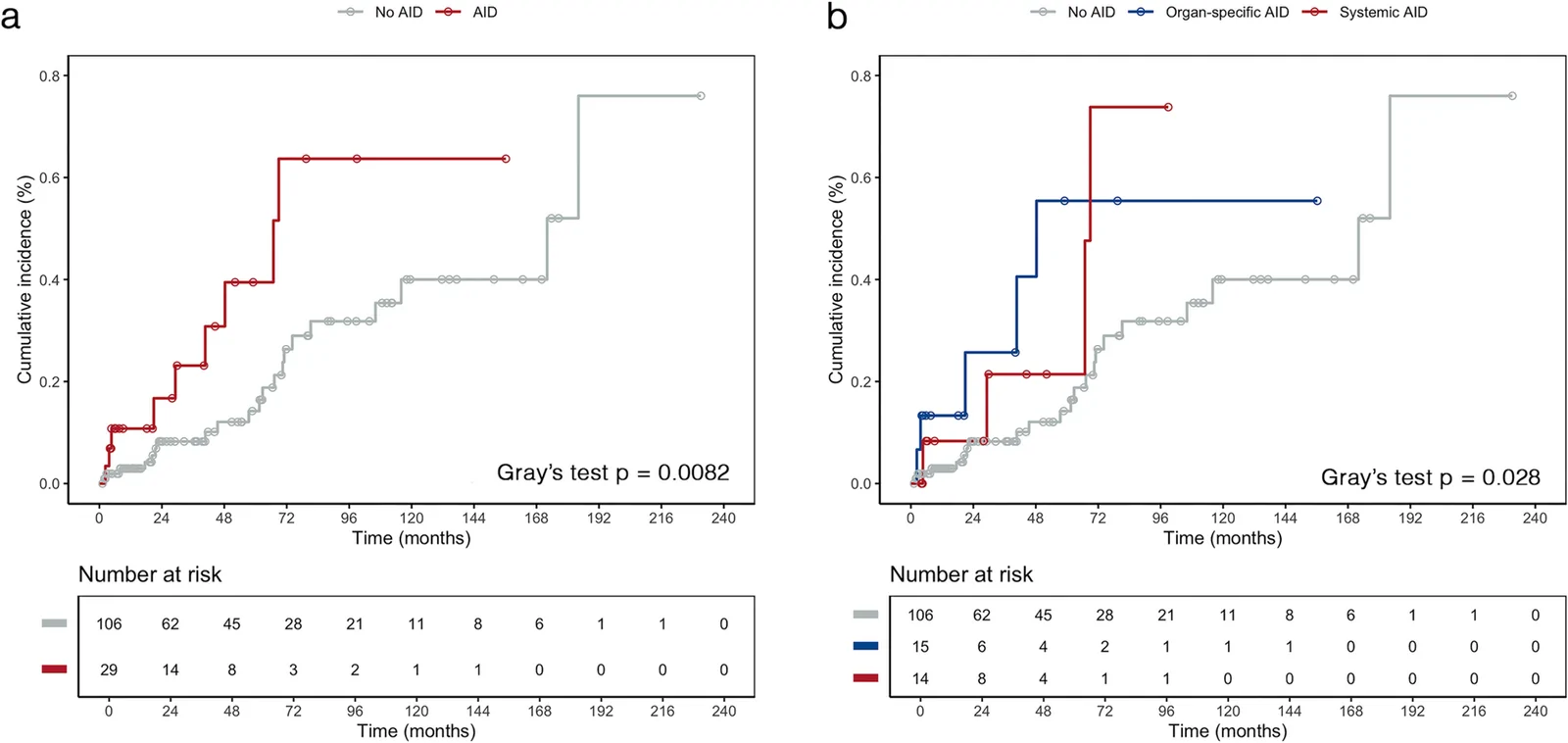
Cumulative incidence of adverse events according to (**a**) AID history and (**b**) subtype in MF patients. (**A**) Patients with a history of AID experienced a significantly higher 3-year cumulative incidence of adverse events [23% (95% CI, 7.5–44)] compared to those without AID [8.2% (95% CI, 3.6–15), *p* = 0.0082]. **(B)** Stratification by AID subtype showed similar elevated cumulative incidences in systemic [21% (95% CI, 2.4–53)] and organ-specific AID [26% (95% CI, 4.6–55)]. Abbreviations: AID, autoimmune disease; MF, myelofibrosis

During follow-up, a total of 45 adverse events were recorded, including 22 cases of infection (16%, of which 14 were grade ≥ 3), 19 cardiovascular events (14%), and 4 malignancies (3%) (**Supplementary Table ****4**). The 3-year cumulative incidence of adverse events was 13% (95% CI, 7.2–20). Event rates were significantly higher in patients with a history of AID (26%) compared to those without AID (9.3%, *p* = 0.0082) (Fig. 2a). When stratified by AID subtype, event rates were comparable across groups (Fig. 2b).

**Fig. 3:**
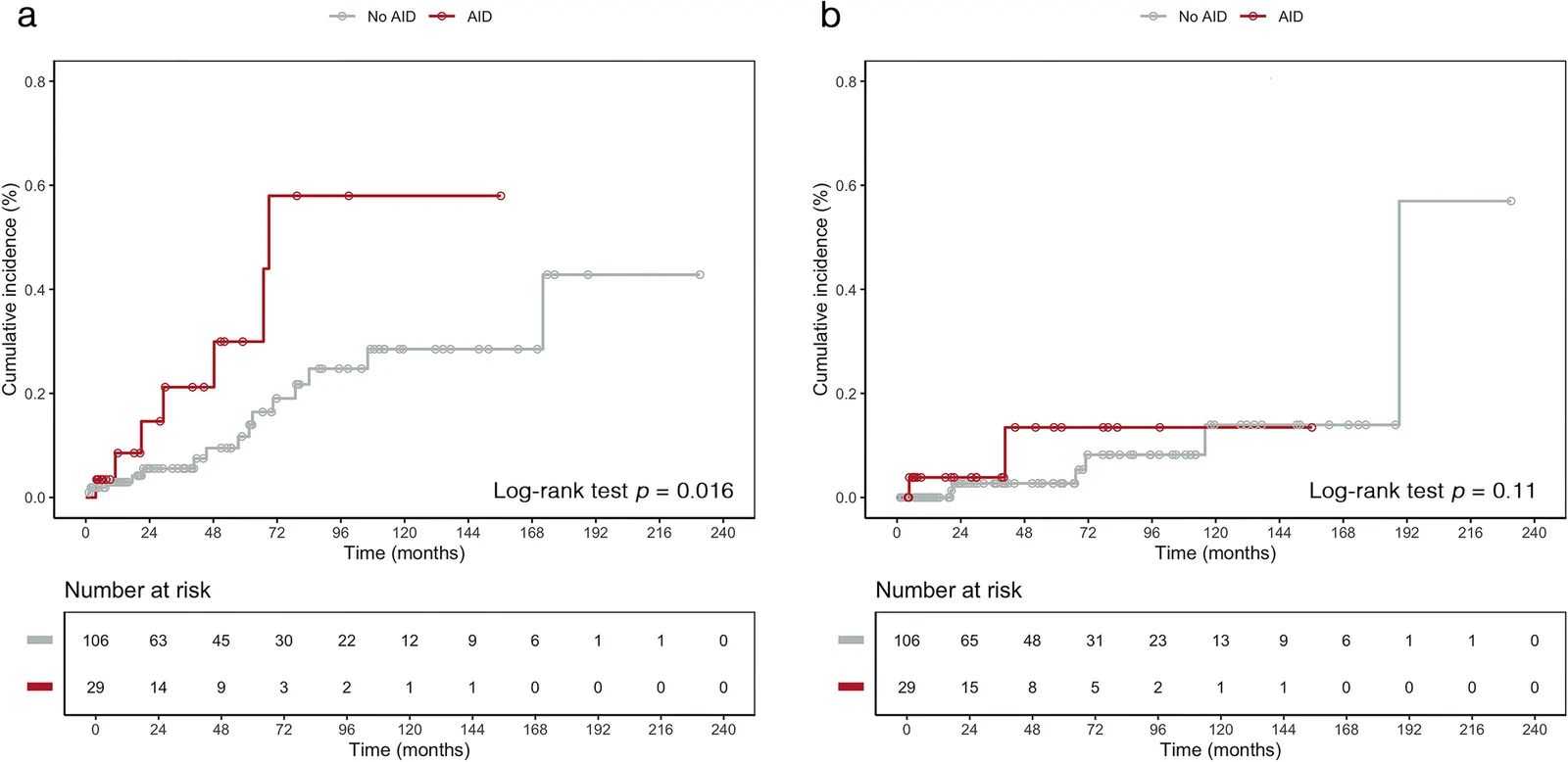
Cumulative incidence of (**a**) infections and (**b**) thrombotic events by AID status in MF patients. (**A**) Infections were significantly more frequent in patients with AID [21% (95% CI, 5.9–43%)] compared to those without AID [5.6% (95% CI, 2.0–12.0), *p* = 0.016]; (**B**) Cardiovascular and thrombotic events occurred more often in the AID group (11%) than in the non-AID group (3.7%), though the difference was not statistically significant (*p* = 0.11). Abbreviations: AID, autoimmune disease; MF, myelofibrosis

Notably, grade ≥ 3 infections were documented in 14 patients (10.4%) and occurred with greater frequency among those with AID. The 3-year cumulative incidence was 21% in patients with AID versus 5.6% in those without (*p* = 0.016; Fig. 3a). This association remained significant after adjustment for age, sex, neutrophil count, corticosteroid exposure, and JAK-inhibitor treatment (HR 3.28; 95% CI, 1.24–8.72, *p* = 0.0169).

Overall, 9 thrombotic events were recorded in 8 patients (5.9%), with a 3-year cumulative incidence that did not differ significantly between the AID cohort [3.7% (95% CI, 0.26–17%)] and the non-AID group [2.7% (95% CI, 0.51–8.5%), *p* = 0.3] (Fig. 3B). Moreover, only two hemorrhagic events were recorded in one patient.

## Discussion

To our knowledge, this is the largest series of patients with MF and concomitant AID reported so far. Our findings provide novel insights into the epidemiology and prognostic implications of AID co-occurrence in MF. Baseline characteristics were largely consistent with previous MF cohorts [20, 21]. However, the 21.5% prevalence of AID observed here markedly exceeds the 2–8% range previously reported in MPN studies [11–13]. While Sardinia’s well-documented genetic predisposition to autoimmunity may partly account for this excess [22], our findings remain notable even in this context. A large population-based study of 25.885 Sardinian adults reported an overall AID prevalence of only 5%, with a female predominance (6.9% vs. 2.8%) [23]. These data are in line with earlier Danish studies [24, 25], yet lower than recent UK data. Indeed, in a national analysis of 22 million UK individuals, the age- and sex-adjusted AID prevalence increased from 7.7% in 2000–2002 to 11% in 2017–2019, reaching 13.1% in women and 7.4% in men [26].

Several methodological and biological factors may account for this difference. First, referral bias inherent to a tertiary oncology center likely enriched our cohort for patients with complex comorbidities. Second, our pragmatic classification encompassed both systemic and organ-specific AID, whereas some prior MPN studies focused predominantly on systemic forms or clinically overt autoimmune manifestations [12]. This inclusive approach may have captured a broader spectrum of autoimmune phenomena, including conditions such as autoimmune thyroiditis that might be underreported in studies using narrower definitions. Finally, a contribution from disease-intrinsic immune dysregulation cannot be excluded. MF is characterized by profound inflammatory dysregulation driven by constitutive JAK-STAT signaling and aberrant cytokine production [27]. This may reflect a bidirectional interplay whereby chronic inflammation driven by the mutant clone sustains clonal expansion while disrupting immune tolerance processes, promoting adaptive immune imbalance and the emergence of autoreactive clones [4].

In line with prior studies [12–14], AID were more frequent in females, whereas no major differences were observed in age at diagnosis, hematologic parameters, comorbidities, or molecular and prognostic profiles, challenging the notion of a distinct clinical phenotype. These findings align with some reports [13, 14], yet contrast with data suggesting younger age and greater splenomegaly in MPN patients with AID [12]. Interestingly, the higher proportion of post-ET MF observed in the AID cohort may suggest a contribution to disease progression of ET. A possible biological explanation is that chronic autoimmune inflammation promotes fibrogenesis or disrupts clonal equilibrium through a sustained cytokine signaling [12]. Still, evidence remains conflicting. While Galimberti et al. [12] described increased fibrotic progression in both PV and ET with a history of AID, Loscocco et al. [28] found no significant association between prior AID and myelofibrosis progression or leukemic transformation in PV/ET, despite observing a higher cumulative incidence of incident AID and second cancers in patients with prior AID. Similarly, Aperna et al. [13] and Elessa et al. [14] reported no significant impact of AID on progression-free survival in large PV and ET cohorts. Thus, the potential role of AID as a biological cofactor in MPN progression remains speculative and warrants prospective validation to delineate whether, and under what circumstances, autoimmunity may alter the natural history of these disorders.

A key finding from our analysis is the reduced survival observed in patients with preexisting systemic AID compared to those with organ-specific forms. This contrasts with prior studies in MPNs and other myeloid neoplasms. In MDS, some reports have even suggested improved survival among patients with concomitant AID [7, 29], while in CMML, AID has shown no prognostic impact [8]. Similarly, in previously studied MPN cohorts, the presence of AID has not been associated with inferior survival [12–14]. In the ET/PV cohort reported by Loscocco et al. [28], prior AID showed only a borderline association with inferior OS, while exploratory analyses suggested that specific inflammatory AID categories, such as inflammatory arthritis, vasculitis, or unclassified AID, may carry a higher adverse prognostic signal than organ-specific conditions. This observation is conceptually consistent with our finding that systemic, rather than organ-specific, AID identifies a clinically vulnerable MF subgroup. A possible explanation for this discrepancy lies in the composition of our cohort, which included only patients with MF. In this setting, systemic autoimmunity, likely reflecting a more profound immune dysregulation, may contribute to a higher burden of comorbidities and an increased incidence of severe infectious events, as also observed in our series.

Among adverse prognostic variables, anemia is a well-established factor included in both the DIPSS and MYSEC prognostic models [16, 17]. Reported in 35–38% of MF patients at diagnosis when defined as hemoglobin < 10 g/dL [16, 30, 31], anemia was present in 40.7% of our cohort, raising the question of whether this higher rate might be related to the elevated AID prevalence. Indeed, hematologic abnormalities, including anemia, are common in systemic AID such as SLE and RA [32–34]. In subgroup analyses, patients with organ-specific AID showed numerically lower hemoglobin levels and higher LDH; however, these differences were not statistically significant, and their mechanistic interpretation was limited by the lack of systematic iron indices, vitamin B12/folate levels, and hemolytic markers. In our analysis, anemia at diagnosis independently predicted inferior survival, and its prognostic impact persisted after adjustment for AID and other covariates. These findings support the notion that anemia in MF predominantly reflects disease-intrinsic hematopoietic impairment, rather than immune-mediated mechanisms linked to AID. New JAK-inhibitors such as momelotinib and pacritinib, inhibiting the ACVR1/ALK2 pathway and the production of hepcidin, favoring iron availability and ameliorating anemia, could play a key role in survival outcome of MF patients with AID [35, 36].

AID have been associated with increased thrombotic risk, with RA, SLE, and Sjögren’s syndrome conferring 2.2- to 7.3-fold higher rates of thrombosis compared to matched controls [37]. Autoimmune cytopenias such as AIHA and ITP also carry an elevated thrombotic risk [38, 39]. In MPN, previous studies have reported a higher incidence of thrombotic events preceding diagnosis in patients with AID, though their presence does not appear to impact thrombotic risk during follow-up [13]. Of note, Krečak et al. [40] specifically addressed this issue in PV and reported that patients with AID at diagnosis experienced more thrombotic events during follow-up and had a significantly shorter time to thrombosis, with the association remaining significant in multivariable analysis. Conversely, in the larger ET/PV cohort by Loscocco et al. [28] prior AID was associated with venous thrombosis before or at MPN diagnosis, but not with arterial or venous thrombosis after diagnosis. Thrombotic and hemorrhagic complications remain clinically relevant in MF, with a recent meta-analysis of 13.436 MPN patients reporting event rates of 9.5% and 8.9%, respectively [41]. In our cohort, thrombotic events were infrequent and not associated with AID status, although cardiovascular and thrombotic events numerically occurred more often among patients with AID. Taken together, these data suggest that the thrombotic impact of AID may differ across MPN phenotypes and may be more apparent in PV, where erythrocytosis, *JAK2*-driven inflammation, and autoimmune vascular injury may converge, than in MF, where competing drivers of morbidity and mortality may predominate.

Infectious complications represent a major source of morbidity and mortality in MF, largely driven by cytopenias and disease-related immune dysfunction [21]. A Swedish population-based study of 8.363 MPN patients reported a 2-fold increased risk of infections across MPNs and nearly 4-fold in PMF, independent of treatment exposure, pointing to intrinsic disease susceptibility [42]. AID such as AIHA, RA, and SLE further amplify infection risk due to both immune dysregulation and immunosuppressive therapies [43–45]. In our cohort, grade ≥ 3 infections occurred more frequently in patients with concomitant AID, and this association persisted after adjusting for age, sex, neutrophil count, and exposure to corticosteroids or JAK-inhibitors. These findings suggest a synergistic vulnerability in this subgroup, underscoring the need for heightened surveillance and tailored infection prevention strategies.

This study has several limitations. Its retrospective, single-center design may limit generalizability and introduce selection bias, including potential geographic or genetic enrichment related to the Sardinian background. In addition, the absence of systematic cytokine profiling, autoantibody assessment, longitudinal immunologic data, and standardized measures of autoimmune disease activity limited mechanistic interpretation and prevented reliable classification of AID as active or controlled at MF diagnosis. The heterogeneity and small number of individual AID subtypes also precluded robust subtype-specific analyses of follow-up events, including infections, thrombosis, cardiovascular events, and secondary malignancies. Moreover, NGS-derived mutational data were available only for a subset of patients, limiting incorporation of molecularly informed MF risk models and assessment of potential associations between AID and CHIP- or myeloid-related mutations. Finally, the relatively limited follow-up warrants caution in interpreting long-term outcomes, including leukemic transformation. Nonetheless, the study benefits from a large and well-characterized MF cohort, the highest reported prevalence of AID in this setting, and granular data on infectious and thrombotic events, enabling a detailed evaluation of their clinical and prognostic relevance.

## Conclusion

In conclusion, this study represents the largest series to date exploring the intersection of MF and AID, revealing an unexpectedly high prevalence of autoimmune comorbidity in this setting. Although clinical and prognostic features were largely comparable, the significantly increased incidence of severe infections among the AID cohort underscores the need for clinical monitoring and individualized management. These findings highlight the complex interplay between immune dysregulation and MF, warranting a multidisciplinary approach. Future prospective studies incorporating comprehensive molecular and immunologic profiling are essential to elucidate the underlying mechanisms and to inform tailored therapeutic strategies.

## Supplementary Information

Below is the link to the electronic supplementary material.

Supplementary Material 1(DOCX 0.98 MB)

## Data Availability

Data and medical charts are available at the Hematology Unit of Businco Hospital, but access to these data is restricted; they were used under licence for the current study and are therefore not publicly available. The data are, however, available upon request and with the permission of the authors.
