# Supplementary material for: Clinical and prognostic significance of concomitant autoimmune disease in patients with myelofibrosis
Source: Ann Hematol. 2026 Jun 26;105(10):412. doi: 10.1007/s00277-026-07128-4 (PMC13572334; doi:10.1007/s00277-026-07128-4)
Supplement: Supplementary file 1 [file 277_2026_7128_MOESM1_ESM.docx]

**CLINICAL AND PROGNOSTIC SIGNIFICANCE OF CONCOMITANT AUTOIMMUNE DISEASE (AID) IN PATIENTS WITH MYELOFIBROSIS**

**Supplementary Fig. S1. Patients’ flowchart.**

Abbreviations: Ab, autoantibody; AID, autoimmune disease; MF, myelofibrosis.

**Supplementary Table 1. STROBE Checklist for case-control studies.**

|  | **Item No** | **Recommendation** | **Page No** |
| --- | --- | --- | --- |
| **Title and abstract** | 1 | (*a*) Indicate the study’s design with a commonly used term in the title or the abstract | 2 |
|  |  | (*b*) Provide in the abstract an informative and balanced summary of what was done and what was found | 2 |
| **Introduction** | | | |
| **Background/rationale** | 2 | Explain the scientific background and rationale for the investigation being reported | 3 |
| **Objectives** | 3 | State specific objectives, including any prespecified hypotheses | 4 |
| **Methods** | | | |
| **Study design** | 4 | Present key elements of study design early in the paper | 4 |
| **Setting** | 5 | Describe the setting, locations, and relevant dates, including periods of recruitment, exposure, follow-up, and data collection | 4 |
| **Participants** | 6 | (*a*) Give the eligibility criteria, and the sources and methods of case ascertainment and control selection. Give the rationale for the choice of cases and controls | 4 |
|  |  | (*b*) For matched studies, give matching criteria and the number of controls per case | 4 |
| **Variables** | 7 | Clearly define all outcomes, exposures, predictors, potential confounders, and effect modifiers. Give diagnostic criteria, if applicable | 4 |
| **Data sources/ measurement** | 8 | For each variable of interest, give sources of data and details of methods of assessment (measurement). Describe comparability of assessment methods if there is more than one group | 4 |
| **Bias** | 9 | Describe any efforts to address potential sources of bias | 4 |
| **Study size** | 10 | Explain how the study size was arrived at | 4 |
| **Quantitative variables** | 11 | Explain how quantitative variables were handled in the analyses. If applicable, describe which groupings were chosen and why | 4 |
| **Statistical methods** | 12 | (*a*) Describe all statistical methods, including those used to control for confounding | 5 |
|  |  | (*b*) Describe any methods used to examine subgroups and interactions | 5 |
|  |  | (*c*) Explain how missing data were addressed | 4 |
|  |  | (*d*) If applicable, explain how matching of cases and controls was addressed | n/a |
|  |  | (*e*) Describe any sensitivity analyses | n/a |
| **Results** | | | |
| **Participants** | 13 | (a) Report numbers of individuals at each stage of study—eg numbers potentially eligible, examined for eligibility, confirmed eligible, included in the study, completing follow-up, and analysed | 5-6 |
|  |  | (b) Give reasons for non-participation at each stage | n/a |
|  |  | (c) Consider use of a flow diagram |  |
| **Descriptive data** | 14 | (a) Give characteristics of study participants (eg demographic, clinical, social) and information on exposures and potential confounders | Supplementary Data, p. 1 |
|  |  | (b) Indicate number of participants with missing data for each variable of interest | 5-7 |
| **Outcome data** | 15 | Report numbers in each exposure category, or summary measures of exposure | 5-7 |
| **Main results** | 16 | (*a*) Give unadjusted estimates and, if applicable, confounder-adjusted estimates and their precision (eg, 95% confidence interval). Make clear which confounders were adjusted for and why they were included | n/a |
|  |  | (*b*) Report category boundaries when continuous variables were categorized | n/a |
|  |  | (*c*) If relevant, consider translating estimates of relative risk into absolute risk for a meaningful time period | n/a |
| **Other analyses** | 17 | Report other analyses done—eg analyses of subgroups and interactions, and sensitivity analyses | 5-8 |
| **Discussion** | | | |
| **Key results** | 18 | Summarise key results with reference to study objectives | 8-9 |
| **Limitations** | 19 | Discuss limitations of the study, taking into account sources of potential bias or imprecision. Discuss both direction and magnitude of any potential bias | 9 |
| **Interpretation** | 20 | Give a cautious overall interpretation of results considering objectives, limitations, multiplicity of analyses, results from similar studies, and other relevant evidence | 8-9 |
| **Generalisability** | 21 | Discuss the generalisability (external validity) of the study results | 8-9 |
| **Other information** | | | |
| **Funding** | 22 | Give the source of funding and the role of the funders for the present study and, if applicable, for the original study on which the present article is based | 11 |

**Supplementary Table 2. Clinical, laboratory, and molecular characteristics of patients with systemic (n = 14) versus organ-specific AID (n = 15).**

| Variables | Systemic AID,  n = 14 | Organ-specific AID,  n= 15 | *p* |
| --- | --- | --- | --- |
| Female sex, n (%) | 7 (50.0) | 11 (73.3) | 0.263 |
| Age, median years (IQR) | 67.5 (63.5-72.0) | 75 (66.0-78.0) | 0.18 |
| PMF, n (%) | 11 (78.6) | 6 (40.0) | 0.060 |
| SMF, n (%) | 3 (21.4) | 9 (60.0) |  |
| Post-ET MF | 3 (21.4) | 8 (53.3) |  |
| Post-PV MF | 0 | 1 (6.7) |  |
| Hb, g/dL, median (IQR) | 11.9 (9.7-13.2) | 9.1 (8.25-10.45) | 0.09 |
| WBCs, x10^9^/L, median (IQR) | 6.43 (3.0-17.9) | 9.18 (7.0-16.4) | 0.429 |
| Hematocrit, % (IQR) | 35.2 (30.6-40.2) | 32.1 (27.4-34.6) | 0.659 |
| PLTs, x10^9^/L, median (IQR) | 289 (115-438) | 371 (232-572) | 0.726 |
| LDH, median U/I (IQR) | 370 (230-999) | 638 (267-821) | 0.976 |
| Palpable splenomegaly, n (%) | 8 (57.1) | 10 (66.7) | 0.710 |
| Driver mutations, n (%) |  |  | 0.643 |
| *JAK2* V617F mut | 7 (50.0) | 10 (66.7) |  |
| *CALR* mut | 4 (28.6) | 3 (20.0) |  |
| *MPL* mut | 0 | 1 (6.7) |  |
| Triple-negative | 3 (21.4) | 1 (6.7) |  |
| DIPSS, n (%) |  |  | 0.569 |
| Low-risk | 0 | 0 |  |
| Intermediate-1 | 3 (21.4) | 3 (20.0) |  |
| Intermediate-2 | 6 (42.9) | 2 (13.3) |  |
| High risk | 3 (21.4) | 1 (6.7) |  |
| MYSEC, n (%) |  |  | 0.670 |
| Low-risk | 1 (7.1) | 0 |  |
| Intermediate-1 | 1 (7.1) | 4 (26.7) |  |
| Intermediate-2 | 1 (7.1) | 4 (26.7) |  |
| High risk | 0 | 1 (6.7) |  |

Abbreviations: AID, autoimmune disease; DIPSS, Dynamic International Prognostic Scoring System; ET, essential thrombocythemia; Hb, hemoglobin; IQR, interquartile range; MYSEC, Myelofibrosis Secondary to PV and ET-Prognostic Model; PLT, platelets; PMF, primary MF; PV, polycythemia vera; SMF, secondary MF; WBC, white blood cells.

**Supplementary Table 3. Treatment patterns at last follow-up in patients with and without AID.**

| Variables | Total, *n* = 135 | No AID,  *n* = 106 | AID,  *n* = 29 | *p* |
| --- | --- | --- | --- | --- |
| First line treatment, n (%) |  |  |  |  |
| Hydroxyurea | 50 (37.0) | 40 (37.7) | 10 (34.5) | 0.917 |
| JAK inhibitor | 50 (37.0) | 41 (38.7) | 9 (31.0) | 0.590 |
| Ruxolitinib | 44 (32.6) | 37 (34.9) | 7 (24.1) | 0.383 |
| Momelotinib | 4 (3.0) | 2 (1.9) | 2 (6.9) | 0.202 |
| Pacritinib | 2 (1.5) | 2 (1.9) | 0 | 1.0 |
| Pipobroman | 2 (1.5) | 2 (1.9) | 0 | 1.0 |
| Danazol | 3 (2.2) | 3 (2.8) | 0 | 1.0 |
| BST | 12 (8.9) | 8 (7.5) | 4 (13.8) | 0.287 |
| No treatment | 15 (11.1) | 11 (10.4) | 4 (13.8) | 0.739 |
| Second line treatment, n (%) | 16 (11.9) | 12 (11.3) | 4 (3.8) | 0.748 |
| JAK inhibitor | 9 (6.7) | 7 (6.6) | 2 (6.9) | 1.0 |
| Ruxolitinib | 4 (3.0) | 4 (3.8) | 0 | 0.577 |
| Momelotinib | 4 (3.0) | 2 (1.9) | 2 (6.9) | 0.202 |
| Fedratinib | 1 (0.7) | 1 (0.9) | 0 | 1.0 |
| BST | 7 (5.2) | 5 (4.7) | 2 (6.9) | 0.643 |
| Third line treatment, n (%) | 3 (2.2) | 3 (2.8) | 0 | 0.643 |
| Danazol | 2 (1.5) | 2 (1.9) | 0 | 1.0 |
| BST | 1 (0.7) | 1 (0.9) | 0 | 1.0 |

Abbreviations: AID, autoimmune disease; BST, best supportive therapy.

**Supplementary Table 4. Detailed adverse events recorded during follow-up.**

| Category | Diagnosis, n (%) | No AID, *n* = 106 | AID, *n* = 29 |
| --- | --- | --- | --- |
| Infections | **Total** | 14 (13.2) | 8 (27.6) |
|  | **Pneumonia** | 8 (7.5) | 4 (13.8) |
|  | **Sepsis** | 2 (1.9) | 2 (6.9) |
|  | **UTI** | 1 (0.9) | 2 (6.9) |
|  | **Viral hepatitis** | 1 (0.9) | 0 |
|  | **Herpes zoster** | 1 (0.9) | 0 |
|  | **Esophageal candidiasis** | 1 (0.9) | 0 |
| Cardiovascular events | **Total** | 13 (12.3) | 6 (20.7) |
|  | **ACS/MI** | 3 (2.8) | 0 |
|  | **Arrhythmias** | 3 (2.8) | 2 (6.9) |
|  | **Pericarditis** | 0 | 2 (6.9) |
|  | **Arterial thrombosis** | 1 (0.9) | 0 |
|  | **DVT/PE** | 2 (1.9) | 2 (6.9) |
|  | **Hemorrhage** | 2 (1.9) | 0 |
|  | **Pulmonary hypertension** | 2 (1.9) | 0 |
| Malignancies | **Total** | 4 (3.8) | 0 |
|  | **Melanoma** | 1 (0.9) | 0 |
|  | **SCC** | 2 (1.9) | 0 |
|  | **Prostate carcinoma** | 1 (0.9) | 0 |

Abbreviations: ACS, acute coronary syndrome; DVT, deep vein thrombosis; MI, myocardial infarction; PE, pulmonary embolism; SCC, squamous cell carcinoma; UTI, urinary tract infections;
